# Anti-*Toxoplasma gondii* antibodies as a risk factor for the prevalence and severity of systemic lupus erythematosus

**DOI:** 10.1186/s13071-024-06141-8

**Published:** 2024-01-30

**Authors:** Zhongzhen Li, Zhiwei Lei, Wanying Yang, Chunxia Jing, Xiaolin Sun, Guang Yang, Xiaozhen Zhao, Mingjiao Zhang, Miaomiao Xu, Yuanjia Tang, Qingwen Wang, Jing Zhao, Zixing Zhou, Zihao Wen, Xiaojing Chen, Qinglin Peng, Guochun Wang, Pingjing Zhang, Erwei Sun, Nan Shen, Weiguo Xu, Zhanguo Li, Hengwen Yang, Zhinan Yin

**Affiliations:** 1 Guangdong Provincial Key Laboratory of Tumor Interventional Diagnosis and Treatment, Zhuhai Institute of Translational Medicine, Zhuhai People’s Hospital (Zhuhai Clinical Medical College of Jinan University), Jinan University, Zhuhai, 519000 China; 2 Department of Basic Medical Research, The Sixth Affiliated Hospital of Guangzhou Medical University, Qingyuan People’s Hospital, Qingyuan, 511518 China; 3 Department of Rheumatology and Immunology, Peking University People’s Hospital and Beijing Key Laboratory for Rheumatism Mechanism and Immune Diagnosis (BZ0135), Beijing, 100034 China; 4 Department of Rheumatology and Immunology, The Third Affiliated Hospital, Southern Medical University, Guangzhou, 510630 China; 5 Department of Epidemiology, School of Medicine, Jinan University, Guangzhou, 510632 China; 6 Shanghai Institute of Rheumatology, Renji Hospital, Shanghai Jiao Tong University School of Medicine, Shanghai, 200001 China; 7 Institute of Clinical Immunology, Academy of Orthopedics Guangdong Province, Guangzhou, 510630 China; 8 Guangdong Key Laboratory of Environmental Pollution and Health, Jinan University, Guangzhou, 510632 China; 9 Department of Rheumatism and Immunology, Peking University Shenzhen Hospital, Shenzhen, 518036 China; 10 Department of Rheumatology, Beijing Key Lab for Immune-Mediated Inflammatory Diseases, China-Japan Friendship Hospital, Beijing, 100029 China; 11 Department of Pathogen Biology, School of Medicine, Jinan University, Guangzhou, 510632 China

**Keywords:** SLE, *Toxoplasma gondii*, Autoantibody, Risk factor

## Abstract

**Background:**

Systemic lupus erythematosus (SLE) is a complex systemic autoimmune disease characterized by the presence of numerous autoantibodies. The interaction of infectious agents (viruses, bacteria and parasites) and a genetically susceptible host may be a key mechanism for SLE. *Toxoplasma gondii* is a widespread intracellular parasite that has been implicated in the pathogenesis of autoimmune diseases. However, the relationship between *T. gondii* infection and the increased risk of SLE in Chinese populations remains unclear.

**Methods:**

The seroprevalence of *T. gondii* infection was assessed in 1771 serum samples collected from Chinese individuals (908 healthy controls and 863 SLE patients) from different regions of China using an enzyme-linked immunosorbent assay. Serum autoantibodies and clinical information were obtained and analysed.

**Results:**

Our observations revealed a higher prevalence of anti-*T. gondii* antibodies (ATxA) immunoglobulin G (IgG) in serum samples from SLE patients (144/863, 16.7%) than in those from the healthy controls (53/917, 5.8%;* P* < 0.0001), indicating a 2.48-fold increased risk of SLE in the ATxA-IgG^+^ population, after adjustment for age and sex (95% confidence interval [CI] 1.70–3.62, *P* < 0.0001). ATxA-IgG^+^ SLE patients also showed a 1.75-fold higher risk of developing moderate and severe lupus symptoms (95% CI 1.14–2.70, *P* = 0.011) compared to ATxA-IgG^−^ patients. Relative to ATxA-IgG^−^ patients, ATxA-IgG^+^ patients were more likely to develop specific clinical symptoms, including discoid rash, oral ulcer, myalgia and alopecia. Seven antibodies, namely anti-ribosomal RNA protein (rRNP), anti-double stranded DNA (dsDNA), anti-cell membrane DNA (cmDNA), anti-scleroderma-70 (Scl-70), anti-cardiolipin (CL), anti-beta2-glycoprotein-I (B2GPI) and rheumatoid factor (RF), occurred more frequently in ATxA-IgG^+^ patients. When combined with anti-dsDNA and RF/anti-rRNP/anti-cmDNA/ESR, ATxA-IgG significantly increased the risk for severe lupus.

**Conclusions:**

Our results suggest that ATxA-IgG may be a significant risk factor for SLE prevalence and severity in Chinese populations.

**Graphical Abstract:**

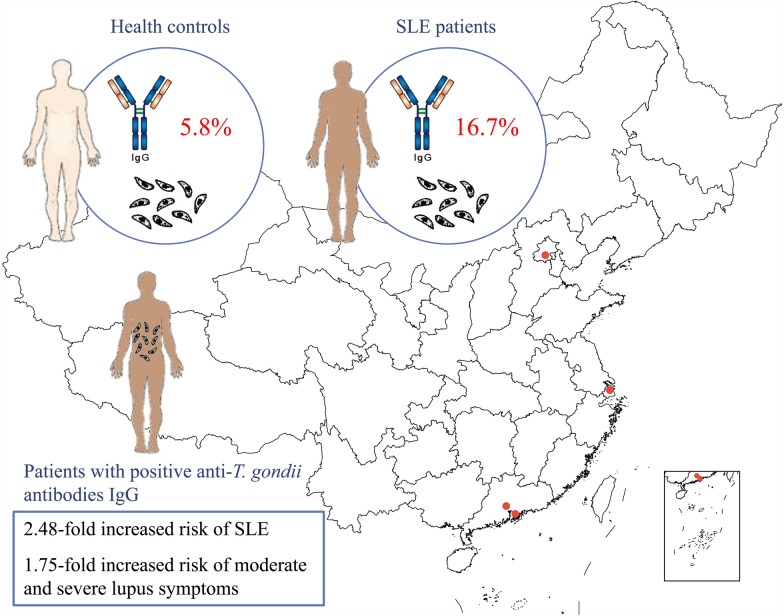

**Supplementary Information:**

The online version contains supplementary material available at 10.1186/s13071-024-06141-8.

## Background

Systemic lupus erythematosus (SLE) is a complex systemic autoimmune disease whose development is determined by both genetic predisposition and exposure to environmental factors such as UV light, drugs, psychological stress and infections [1–6]. This autoimmune disorder is characterized by the production of multiple autoantibodies, diverse clinical manifestations and the presence of anti-nuclear antibodies [7–9]. In patients with SLE, the polyreactive B cells produce a range of autoantibodies, including anti-double stranded DNA (dsDNA), anti-Smith (Sm), anti-ribonucleoprotein (RNP), anti-Ro, anti-La, anti-phospholipid and anti-nuclear antigen (ANA) antibodies [10]. Of these seven autoantibodies, the anti-Sm and anti-dsDNA antibodies are specific to SLE and have been reported to participate in immune complex formation and inflammatory damage to multiple end-organs, such as the kidney, skin and central nervous system [10, 11]. Researchers on SLE have also become interested in the platelet-mediated release of mitochondrial DNA, which is a additional source of nucleic acids, in SLE, such as rheumatoid factor (RF) [12, 13]. While the etiology and pathogenesis of SLE remain largely unknown, conventional assays for ANA, anti-dsDNA and other autoantibodies are usually performed for the diagnosis, monitoring and treatment of SLE [14, 15].

Toxoplasmosis is a parasitic infection caused by the obligate intracellular protozoan *Toxoplasma gondii*. This parasite can infect nearly all warm-blooded animals, including humans, and it has been reported that one third of all humans are infected, with large differences between countries (from 4% to 60%) [16–18]. The most common symptoms of toxoplasmosis in humans is lymphadenopathy, which may be associated with a sore throat, fever, fatigue, headache and muscle pain [19, 20]. In most immunocompetent individuals, *T. gondii* infection is asymptomatic. However, in immunocompromised individuals, patients undergoing immunosuppressive treatments or pregnant women, *T. gondii* infection can cause serious clinical symptoms and even death [16, 17, 21].

In humans, infection by parasites, viruses or bacteria frequently induces autoantibodies in the infected individual, which are most commonly associated with autoimmune disorders [22]. The authors of a previous study reported a correlation between *Toxoplasma* antibodies (ATxA) in patients with autoimmune diseases and serum anti-centromere antibodies, such as anti-cardiolipin (CL), anti-beta2-glycoprotein-I (B2GPI), complex anti-cardiolipin-beta2-glycoprotein-I complex (anti-CL-B2) antibodies, anti-gliadin, anti-phosphatidylethanolamine (PE), anti-prothrombin (PT) and anti-scleroderma-70 (anti-Scl-70) [18]. Among these, RF antibodies are typically associated with disease activity and inflammation in rheumatoid arthritis (RA) [23]. In up to 66% of patients with Sjögren syndrome (SS), the presence of ANA, RF, anti-Sjögren’s syndrome A (SSA) and anti-Sjögren’s syndrome B (SSB) antibodies can be detected years before symptom onset [24, 25]. Additionally, anti-nucleosome antigen (ANUA), anti-dsDNA, Sm and SSA antibodies are detected at a high frequency in SLE patients [26]. However, further studies are needed to investigate the link between *T. gondii* infection and autoantibodies in various autoimmune diseases.

*Toxoplasma gondii* has been previously reported to be associated with SLE, as high titres of *Toxoplasma* antibodies were found to be significantly more common in patients with SLE [27]. However, in European populations, anti-*T. gondii* antibodies immunoglobulin G (ATxA-IgG)-positive (ATxA-IgG^+^) individuals have been found to have a higher prevalence of RA but not SLE and SS [18]. Similarly, the prevalence of ATxA-IgG was significantly higher in arthritic patients than in healthy controls in eastern China [28]. However, the association between *T. gondii* infection and increased risk of SLE remains unknown in Chinese populations.

Given the high prevalence of *T. gondii* infection and its possible associations with SLE, we sought to evaluate the seroprevalence of ATxA-IgG in a large cohort of Chinese SLE patients, as well as the risk factors associated with *T. gondii* infection.

## Methods

### Patients and serum samples

Serum samples were collected from 1233 patients with various autoimmune diseases (AIDs) and 908 heathy controls from different regions of China (Beijing, Shanghai, Guangzhou and Shenzhen). The serum samples from the patients with AID included those from 863 SLE patients, 151 RA patients and 219 SS patients. Control serum samples were from healthy subjects of similar age and sex distribution as the patients (300 from northern China, 41 from middle China and 567 from southern China). All serum samples from patients were collected by a clinician following diagnosis. Samples were kept at − 80 °C until analysis.

Information on demographics, such as gender, age, area of residence and ethnicity, was obtained from the computerized inpatient case registry for all patients or was requested from the control individuals themselves. Clinical information, including clinical history, physical examination and laboratory results, was obtained for all clinical cases. All personal information was anonymized and treated as strictly confidential. The discriminant analysis (distinguishing clearly active vs mildly/nonactive disease) of SLE was assessed considering the modified Systemic Lupus Erythematosus Disease Activity Index 2000 (SLEDAI-2K) as the gold standard [29]. The study was approved by the local ethics committees and fulfilled the ethical guidelines of the most recent Declaration of Helsinki (1978, revised 2008).

### Serological testing

Serum samples were analysed for the presence of anti-*T. gondii* IgG antibodies using commercially available enzyme-linked immunosorbent assays (ELISA) kits (Haitai Biotech, Inc., Zhuhai, China). Autoantibodies were assessed using the Bio-Plex 200 immunoassay multiplex array system (Bio-Rad Laboratories, Hercules, CA, USA) according to the manufacturer’s protocol. The autoantibodies included: anti-ANA, ANUA, RF, anti-ribosomal RNA protein (anti-rRNP), anti-dsDNA, anti-Sm, anti-Ro/SSA, anti-La/SSB, anti-Scl-70, anti-CL and anti-B2GPI.

### Statistical analysis

Statistical analysis was performed using SPSS v24.0 statistical software (SPSS IBM, Armonk, NY, USA. Student’s t-test or Chi-square test (*χ*^2^) was used to examine differences in the demographic characteristics and *T. gondii* infection status. Multivariate logistic regression models were used to adjust for potential confounders. Variables associated with *T. gondii* infection were identified by univariate analysis (*P* ≤ 0.05) and included in the multivariate logistic regression analysis. Odds ratios (ORs) and the corresponding 95% confidence intervals (CIs) were calculated to identify independent risk factors for *T. gondii* infection. *P* < 0.05 was considered to indicate statistical significance. The interaction effects were determined on the additive scale, with three measures used to examine biological interaction: (i) attributable proportion due to interaction (AP); (ii) relative excess risk due to interaction (RERI); and (iii) synergy index (S), with S > 1 indicating synergetic effects and S < 1 indicating antagonistic effects [30, 31].

## Results

Serum samples from 1233 AID patients (863 SLE patients, 151 RA patients and 219 SS patients) and 908 healthy controls from different regions of China were included in this study (Additional file 1: Table S1). We observed that the *T. gondii* infection rate was significantly higher in SLE patients (144/863, 16.7%) than in the healthy controls (53/908, 5.8%; *P* < 0.0001) and significantly higher in patients with SS (33/219, 15.1%; *P* < 0.0001) than in the healthy controls (Table 1). In contrast, the infection rate of *T. gondii* in the RA population (12/151, 8.0%, *P* = 0.319) was not significantly different from that in the healthy controls. Although an earlier study in European populations did not find a higher prevalence of ATxA in patients with SLE [18], we believed it worthwhile to evaluate the association between SLE and *T. gondii* infection in Chinese populations, given the large SLE cohort in our sample pool and their high seroprevalence of *T. gondii*.

**Table 1:** Prevalence of anti-*Toxoplasma gondii* antibodies immunoglobulin G in serum samples of patients with different autoimmune diseases and healthy controls

Study population	ATxA IgG^+^	*P* value^a^
*Healthy controls*	53/908 (5.8%)	
*Patients with an AID*		
Rheumatoid arthritis	12/151 (8.0%)	0.319
Sjögren’s syndrome	33/219 (15.1%)	< 0.0001*
Systemic lupus erythematosus	144/863 (16.7%)	< 0.0001*

*AID* Autoimmune disease,* ATxA IgG*^*+*^, anti-*Toxoplasma gondii* antibodies immunoglobulin G-positive,* ATxA IgG*^*−*^ anti-*Toxoplasma gondii* antibodies immunoglobulin G-negative

*Statistically significant

^a^*P* value for the comparison with matched healthy controls

Risk factor analysis demonstrated a 2.48-fold higher risk of SLE in the ATxA-IgG^+^ patient population, after adjustment for age and sex (95% CI 1.70–3.62, *P* < 0.0001) (Table 2). *Toxoplasms gondii* infection was also significantly correlated with disease severity. In comparison with ATxA-IgG^−^ SLE patients, ATxA-IgG^+^ SLE patients were 1.75-fold more likely to develop moderate and severe lupus symptoms (SLEDAI-2 K ≥ 10, 95% CI 1.14–2.70,* P* = 0.011) (Table 3). ATxA-IgG^+^ patients also showed higher frequencies of certain clinical symptoms, such as discoid rash, oral ulcer, myalgia and alopecia (Additional file 2: Table S2).

**Table 2:** Higher risk of systemic lupus erythematosus in anti-*Toxoplasma gondii* antibodies immunoglobulin G population

ATxA-IgG type	Healthy controls (*N*, %)	SLE patients (*N*, %)	Odds ratio (95% CI)	Odds ratio (95% CI) (age/sex adjusted)	*P*-value^a^
ATxA-IgG^−^	855 (94.2)	719 (83.3)	Reference	Reference	
ATxA IgG^+^	53 (5.8)	144 (16.7)	3.23 (2.32–4.49)	2.48 (1.70–3.62)	< 0.0001*

* ATxA IgG*^*+*^, Anti-*Toxoplasma gondii* antibodies immunoglobulin G-positive,* ATxA IgG*^*−*^ anti-*Toxoplasma gondii* antibodies immunoglobulin G-negative,* CI* confidence interval

*Statistically significant

^a^*P* value for the comparison with matched healthy controls

**Table 3:** Associations of anti-*T. gondii* antibodies immunoglobulin G with disease severity, but not duration

Disease activity and duration	Odds ratio	95% CI	*P* value^a^
*Disease activity*			
Low activity (SLEDAI-2 K: < 10)	1.0		
Moderate & Severe activity (SLEDAI-2 K: ≥ 10)	1.75	1.14–2.70	0.011*
*Disease duration*			
< 5 years	1.0		
5–10 years	0.82	0.51–1.30	0.40
≥ 10 years	1.57	0.85–2.90	0.150

* CI* Confidence interval,* SLEDAI-2K* Systemic Lupus Erythematosus Disease Activity Index 2000

*Statistically significant

^a^*P* value: adjusted for sex and age (≤ 40 and > 40 years)

Autoantibodies are considered hallmarks of SLE and are closely associated with disease progression [10]. To confirm whether ATxA-IgG increased the production of any autoantibodies related to lupus severity, we determined the presence or absence of autoantibodies present in the serum of SLE patients and compared the prevalence of these antibodies in ATxA-IgG^+^ and ATxA-IgG^−^ patients. We found that ATxA-IgG^+^ patients were likely to produce more types of autoantibodies, such as anti-rRNP, anti-dsDNA, anti-cmDNA, anti-Scl-70, anti-CL, anti-B2GP1 and RF antibodies (Table 4). Among these autoantibodies, anti-rRNP, anti-dsDNA and anti-cmDNA, which are highly associated with SLE disease development, were significantly elevated in ATxA-IgG^+^ patients (*P* = 0.016, *P* = 0.011 and *P* = 0.00011, respectively; Table 4).

**Table 4:** Autoantibody prevalence in serum from patients with systemic lupus erythematosus in relation to anti-*Toxoplasma gondii* antibodies immunoglobulin G

Autoantibodies^a^	All patients (*n* = 863)	ATxA-IgG^+^ SLE patients (*n* = 144)	ATxA-IgG^−^ SLE patients (*n* = 719)	*P* value^b^
ANA positive, *n* (%)	669 (88.5%)	107 (88.4%)	562 (88.5%)	0.981
ANUA positive, *n* (%)	417 (54.4%)	76 (62.3%)	341 (53.0%)	0.057
Anti-rRNP positive, *n* (%)	246 (37.3%)	51 (47.7%)	195 (35.3%)	0.016*
Anti-Ro/SSA positive, *n* (%)	349 (46.7%)	59 (49.6%)	290 (46.2%)	0.495
Anti-La/SSB positive, *n* (%)	87 (11.6%)	14 (15.9%)	73 (11.6%)	0.960
Anti-dsDNA positive, *n* (%)	518 (65.4%)	97 (75.2%)	421 (63.5%)	0.011*
Anti-cmDNA positive, *n* (%)	33 (5.4%)	13 (13.5%)	20 (3.9%)	0.00011*
Anti-Sm positive, *n* (%)	142 (19.1%)	29 (19.1%)	113 (18.1%)	0.113
Anti-Scl-70, *n* (%)	22 (4.4%)	7 (9.5%)	15 (3.5%)	0.021*
Rheumatoid factor (RF), *n* (%)	93 (12.9%)	22 (19.0%)	71 (11.8%)	0.035*
Anti-CL, *n* (%)	134 (19.2%)	36 (32.4%)	98 (16.7%)	0.00011*
Anti-B2GPI, *n* (%)	78 (11.1%)	18 (16.8%)	60 (10.1%)	0.041*

* ATxA IgG*^*+*^, Anti-*Toxoplasma gondii* antibodies immunoglobulin G-positive,* ATxA IgG*^*−*^ anti-*Toxoplasma gondii* antibodies immunoglobulin G-negative,* SLE* systemic lupus erythematosus

*Statistically significant

^a^ANA, Anti-nuclear antigen; ANUA, anti-nucleosome antigen; rRNP, ribosomal RNA protein, Ro/SSA, Ro/Sjögren’s syndrome A; La/SSB, La/Sjögren’s syndrome B; dsDNA, double-stranded DNA; cmDNA, cell membrane DNA; Sm, Smith; Scl-70, scleroderma-70; CL, cardiolipin; B2GPI, beta2-glycoprotein-I

^b^*P* value: adjusted for age(years) and sex

The interaction of *T. gondii* infection and serum autoantibody levels were also analysed. Based on the S index, there were eight significant synergetic interactions, including erythrocyte sedimentation rate (ESR), D-dimer, RF, anti-streptolysin O (ASO), anti-dsDNA, anti-cmDNA, anti-rRNP and anti-CL antibodies (Additional file 3: Table S3). When combined with anti-dsDNA and RF elements, ATxA-IgG increased the risk for severe lupus by 5.7-fold (OR=14.34 [95% CI 1.85–111.19, *P* = 0.011] vs OR=8.66 [95% CI 2.95–25.43, *P* < 0.0001]; Additional file 4: Table S4). Similarly, ATxA-IgG combined with anti-dsDNA and anti-rRNP increased the lupus severity risk by 1.92-fold (OR=9.00 [95% CI 3.27–29.95, *P* < 0.0001] vs OR=7.08 [95% CI 3.96–12.65, *P* < 0.0001]; Additional file 5: Table S5). Moreover, ATxA-IgG also significantly increased the lupus risk in combination with either anti-dsDNA and ESR (Additional file 6: Table S6) or anti-dsDNA and anti-cmDNA (Additional file 7: Table S7).

## Discussion

Systemic lupus erythematosus is a complex systemic autoimmune disease with an unclear etiology. Previous studies have suggested a potential association between *T. gondii* infection and SLE [18, 27], but the association between *T. gondii* infection and increased risk of SLE remains unknown in Chinese populations. In this study, we sought to evaluate the seroprevalence of anti-*T. gondii* IgG in a large cohort of Chinese SLE patients and identify the risk factors associated with *T. gondii* infection.

To our knowledge, this study is the first nationwide clinical study which involves an epidemiological investigation of *T. gondii* infection in Chinese patients with SLE. We measured ATxA-IgG in a large group of people from different geographic regions of China (Beijing, Shanghai, Guangzhou and Shenzhen). Our results show there was a higher prevalence of ATxA-IgG in our Chinese patients with SLE or SS than in the healthy Chinese controls. Similarly, high titres of *Toxoplasma* antibodies were significantly more common in SLE patients (*n* = 50) [27]. However, the authors of a study involving European populations reported a higher incidence of RS (27/35, 77%) but not SLE (54/169, 32%) in ATxA-IgG^+^ individuals [18]. In this same study, the frequency of ATxA-IgG in sera collected from Latin American patients with RA (55/152, 36%), SLE (42/120, 35%) and SS (33/82, 40%) was similar to that in the healthy controls [18]. A reasonable explanation for these contradictory findings is that individual susceptibility to SLE is highly associated with race, genetic lineage, lifestyle and environmental factors, which include socioeconomic status, dietary habits, exposure to environmental pollutants and infectious agents (either triggering or protective agents). The associations between ATxA-IgG and these factors are likely to contribute to the development of AID. The study [18] included a relatively small number of SLE patients (120 from Latin America and 169 from Europe), which may have limited the ability to fully capture the actual epidemiological mechanism of the disease.

Our data also report an association between ATxA-IgG and the level of serum autoantibodies or ESR, a clinical hallmark of rhupus patients. When combined with anti-dsDNA and RF/anti-rRNP/anti-cmDNA/ESR, ATxA-IgG significantly increased the risk for severe lupus. Anti-dsDNA antibodies, which are specific to SLE (70–80% positive rate), are included in the diagnostic criteria outlined by the American College of Rheumatology. They have been reported to participate in immune complex formation and inflammatory damage to multiple end organs, such as the kidney, skin and central nervous system [10, 11, 32]. RFs play a critical role in the differential diagnosis of polyarthritis and are frequently detected in patients with systemic autoimmune diseases, such as SLE (15–30% positive rate), mixed connective tissue disease, polymyositis and dermatomyositis [13]. Anti-rRNP antibodies are closely linked to severe thrombocytopenia and malar rash, which occur mostly in SLE patients [33, 34]. Anti-cmDNA antibodies could be an efficient diagnostic biomarker for SLE due to their high serum levels and diagnostic specificity, especially for SLE patients who test negative for anti-dsDNA or/and anti-Sm antibodies [35].

Thus, this study presents a new perspective for SLE disease management, as ATxA-IgG might be a significant risk factor for the prevalence and severity of SLE in the Chinese population. Moreover, *T. gondii* infection might produce a higher risk for developing moderate and severe lupus symptoms. However, more research is needed before ATxA-IgG testing can be performed as a routine clinical diagnostic test for SLE, similar to those carried out for autoantibodies.

In SLE disease management, diagnostic accuracy and the appropriate therapy are critical in the clinical setting and for research purposes [36]. Our study results were validated in the following ways. First, our *T. gondii* infection and SLE diagnoses were performed as accurately as possible. All *T. gondii* infection diagnoses were validated using anti-*T. gondii* IgG ELISA kits, which are commonly used for clinical diagnosis in China. Second, the collection of diagnostic and clinical data on SLE patients was performed by experienced clinicians, and all clinical data can be traced back to records in the hospital medical record system. Third, the sample and clinical data were collected from various geographic regions of China, effectively minimizing the impact of regional differences. Finally, we analysed clinical risk factor covariates using a multivariate regression model, which was highly effective in identifying the risk of each element.

However, there are some potential limitations in our study. First, our work did not explore the relationships among *T. gondii* infection, SLE treatment and disease prognosis. Second, we failed to accurately monitor the exact therapeutic regimen and disease prognosis of each patient, due to the diversity and complexity of patient disease progression and treatment. Third, our research did not look at the impact of anti-*T. gondii* immunoglobulin M (IgM) status on SLE disease progression and diagnosis. Thus, a larger and more comprehensive study is required to address this issue. Finally, there is a possibility that the autoantibodies in SLE patients can cross-react with *T. gondii* antigens. It is difficult to obtain a solid evidence, such as, for example, *T. gondii* gene PCR product from the samples, to exclude the possibility of cross-reaction. However, if the seroprevalence in SLE patients was due to a cross-reaction, a much higher serological positive rate—and the observed 16.7%—would be expected. Moreover, a cross-reaction usually results in a low antibody titre. In our study, the ATxA-IgG level was low (slightly above the cutoff point according to the ELISA kit) in 17 out of 144 (11.8%) ATxA-IgG^+^ SLE patients and in seven out of 53 (13.2%) of ATxA-IgG^+^ healthy controls. The similarity of these percentages indicate that both sets of patients have similar ATxA-IgG levels.

## Conclusions

In summary, our study provides new epidemiological evidence on the important role of *T. gondii* infection in SLE disease and further insights into these interactions in SLE etiology. Our results also provide additional evidence that *T. gondii* infection should be considered in SLE disease, especially in Chinese patients.

### Supplementary Information

**Additional file 1: Table S1.** The number of serum samples of patients with different autoimmune diseases and healthy controls.

**Additional file 2: Table S2.** The demographics and disease characteristics of SLE patients.

**Additional file 3: Table S3.** The anti-*T. gondii* antibodies IgG combined with biochemical indexes/autoantibodies for the prediction of SLE.

**Additional file 4: Table S4.** Risk factors for disease severity (analysis with 3 factors): anti*-T. gondii *antibodies IgG, anti-dsDNA and RF.

**Additional file 5: Table S5.** Risk factors for disease severity (analysis with 3 factors): anti-*T. gondii* antibodies IgG, anti-dsDNA and anti-rRNP.

**Additional file 6: Table S6.** Risk factors for disease severity (analysis with 3 factors): anti-*T. gondii* antibodies IgG, anti-dsDNA and ESR.

**Additional file 7: Table S7.** Risk factors for disease severity (analysis with 3 factors): anti-*T. gondii* antibodies IgG, anti-dsDNA and anti-cmDNA.

## Data Availability

All data and materials of the experiments described here are included in this published article and its additional files.

## Abbreviations

AIDs: Autoimmune diseases
ANA: Anti-nuclear antigen
Anti-CL-B2: Complex anti-cardiolipin-Beta2-glycoprotein-I antibodies
ANUA: Anti-nucleosome antigen
ATxA: Anti-*Toxoplasma* antibodies
ATxA-IgG: Anti-*Toxoplasma gondii* antibodies immunoglobulin G
ATxA-IgG^+^: Anti-*Toxoplasma gondii* antibodies immunoglobulin G-positive
ATxA-IgG^−^: Anti-*Toxoplasma gondii* antibodies immunoglobulin G-negative
B2GPI: Beta2-glycoprotein-I
CI: Confidence interval
CL: Cardiolipin
cmDNA: Cell membrane DNA
dsDNA: Double-stranded DNA
ELISA: Enzyme-linked immunosorbent assay
ESR: Erythrocyte sedimentation rate
HFF: Human foreskin fibroblast
IgG: Immunoglobulin G
IgM: Immunoglobulin M
PE: Phosphatidylethanolamine
PT: Anti-prothrombin
RA: Rheumatoid arthritis
RF: Rheumatoid factor
RNP: Ribonucleoprotein
rRNP: Ribosomal RNA protein
Scl-70: Scleroderma-70
SLE: Systemic lupus erythematosus
Sm: Smith
SS: Sjögren syndrome
SSA: Sjögren’s syndrome A
SSB: Sjögren’s syndrome B
