# Supplementary material for: Anti-Toxoplasma gondii antibodies as a risk factor for the prevalence and severity of systemic lupus erythematosus
Source: Parasit Vectors. 2024 Jan 30;17:44. doi: 10.1186/s13071-024-06141-8 (PMC10826107; doi:10.1186/s13071-024-06141-8)
Supplement: Supplementary file 1 — Additional file 1: Table S1. The number of serum samples of patients with different autoimmune diseases and healthy controls. [file 13071_2024_6141_MOESM1_ESM.docx]

**Table S1** The number of serum samples of patients with different autoimmune diseases and healthy controls.

| Diseases | Beijing | Shanghai | Guangzhou | Shenzhen | Total |
| --- | --- | --- | --- | --- | --- |
| Health control | 300 | 41 | 567 | 0 | 907 |
| Rheumatoid arthritis | 100 | 51 | 0 | 0 | 151 |
| Sjögren's syndrome | 219 | 0 | 0 | 0 | 219 |
| Systemic lupus erythematosus | 412 | 271 | 30 | 150 | 863 |
| Total | 1031 | 363 | 596 | 150 | 2140 |
