# Supplementary material for: Anti-Toxoplasma gondii antibodies as a risk factor for the prevalence and severity of systemic lupus erythematosus
Source: Parasit Vectors. 2024 Jan 30;17:44. doi: 10.1186/s13071-024-06141-8 (PMC10826107; doi:10.1186/s13071-024-06141-8)
Supplement: Supplementary file 2 — Additional file 2: Table S2. The demographics and disease characteristics of SLE patients. [file 13071_2024_6141_MOESM2_ESM.docx]

**Table 2** The demographics and disease characteristics of SLE patients.

|  | ALL patients  (n=863) | ATxA-IgG^+^ (n=144) | ATxA-IgG^-^ (n=719) | ^a^*P* value |
| --- | --- | --- | --- | --- |
| **Demographics** |  |  |  |  |
| Age (years), mean (SD), n | 36.3±14.4(n=856) | 32.9±13.9(n=714) | 37.0±14.5(n=142) | 0.002* |
| Female, n (%) | 779 (90.4%) | 130 (90.9%) | 649 (90.3%) | 0.811 |
| **Clinical details** |  |  |  |  |
| Disease duration (years),  mean (SD), n | 6.6±7.1(n=829) | 6.8±7.3(n=136) | 5.2±5.6(n=693) | 0.015* |
| SLEDAI-2K, mean (SD), n | 12.6±6.5(n=823) | 14.1±6.2(n=136) | 12.26±6.5(n=687) | 0.003* |
| Spontaneous abortion, n (%) | 91(12.9%) | 17(13.9%) | 74(12.7%) | 0.720 |
| Hypertension, n (%) | 117(14.1%) | 14(10.4%) | 103(14.9%) | 0.169 |
| Diabetes, n (%) | 57(6.9%) | 6(4.4%) | 51(7.4%) | 0.216 |
| Nephritis, n (%) | 291(35.3%) | 46(33.8%) | 245(35.6%) | 0.699 |
| Malar rash, n (%) | 379(44.7%) | 65(46.4%) | 314(44.4%) | 0.651 |
| Discoid rash, n (%) | 119(14.3%) | 34(24.3%) | 85(12.3%) | 0.0002* |
| Photosensitivity, n (%) | 145(17.1%) | 26(18.6%) | 119(16.8%) | 0.618 |
| Raynaud's phenomenon, n (%) | 108(12.8%) | 23(16.3%) | 85(12.0%) | 0.165 |
| Oral ulcer, n (%) | 181(21.3%) | 40(28.4%) | 141(19.9%) | 0.026* |
| Myosalgia, n (%) | 76(9.0%) | 22(15.7%) | 54(7.7%) | 0.002* |
| Alopecia, n (%) | 273(32.2%) | 63(44.7%) | 210(29.7%) | 0.001* |
| Proteinuria, n (%) | 414(49.6%) | 61(45.2%) | 353(50.4%) | 0.265 |

^a^*P* value: Adjusted for age(years) and sex.

* Statistically significant.
