# Supplementary material for: Anti-Toxoplasma gondii antibodies as a risk factor for the prevalence and severity of systemic lupus erythematosus
Source: Parasit Vectors. 2024 Jan 30;17:44. doi: 10.1186/s13071-024-06141-8 (PMC10826107; doi:10.1186/s13071-024-06141-8)
Supplement: Supplementary file 3 — Additional file 3: Table S3. The anti-T. gondii antibodies IgG combined with biochemical indexes/autoantibodies for the prediction of SLE. [file 13071_2024_6141_MOESM3_ESM.docx]

**Table 3** The anti-*T. gondii* antibodies IgG combined with biochemical indexes/ autoantibodies for the prediction of SLE.

| Interaction group | | Deviation from additive model | |
| --- | --- | --- | --- |
|  | Predictor | S(95%CI) | ^a^*P* value |
| 1 | ESR & ATxA-IgGATxA-IgG | 1.95(1.26-3.03) | 0.003* |
| 2 | D-dimmer & ATxA-IgG | 2.46(1.61-3.74) | <0.0001* |
| 3 | RF & ATxA-IgG | 1.76(1.03-3.00) | 0.038* |
| 4 | Anti-ASO & ATxA-IgG | 2.26(1.09-4.71) | 0.029* |
| 5 | CRP & ATxA-IgG | 1.26(0.82-1.93) | 0.298 |
| 6 | Anti-dsDNA & ATxA-IgG | 1.66(1.06-2.61) | 0.027* |
| 7 | ANUA & ATxA-IgG | 1.40(0.93-2.12) | 0.11 |
| 8 | Anti-Ro/SSA & ATxA-IgG | 1.06(0.71-1.60) | 0.768 |
| 9 | Anti-La/SSB & ATxA-IgG | 0.92(0.49-1.73) | 0.788 |
| 10 | Anti-ANA & ATxA-IgG | 0.90(0.49-1.68) | 0.744 |
| 11 | Anti-Sm & ATxA-IgG | 1.34(0.83-2.18) | 0.234 |
| 12 | Anti-Scl-70 & ATxA-IgG | 2.51(0.91-6.87) | 0.074 |
| 13 | Anti-cmDNA & ATxA-IgG | 3.86(1.84-8.11) | <0.0001* |
| 14 | Anti-rRNP & ATxA-IgG | 1.55(1.00-2.41) | 0.049* |
| 15 | Anti-U1RNP & ATxA-IgG | 1.32(0.87-2.00) | 0.188 |
| 16 | Anti-CL & ATxA-IgG | 2.42(1.53-3.83) | <0.0001* |
| 17 | Anti-β2GPI1 & ATxA-IgG | 1.79(1.00-3.20) | 0.051 |

95% CI: 95% Confidence Interval.

^a^*P* value: Adjusted for age(years) and sex.

* Statistically significant.
