# Supplementary material for: Anti-Toxoplasma gondii antibodies as a risk factor for the prevalence and severity of systemic lupus erythematosus
Source: Parasit Vectors. 2024 Jan 30;17:44. doi: 10.1186/s13071-024-06141-8 (PMC10826107; doi:10.1186/s13071-024-06141-8)
Supplement: Supplementary file 4 — Additional file 4: Table S4. Risk factors for disease severity (analysis with 3 factors): anti-T. gondii antibodies IgG, anti-dsDNA and RF. [file 13071_2024_6141_MOESM4_ESM.docx]

**Table 4** Risk factors for disease severity (analysis with 3 factors): Anti-*T. gondii* antibodies IgG, Anti-dsDNA and RF.

| ATxA-IgG | Anti-dsDNA | RF | OR（95%CI） | ^a^*P value* |
| --- | --- | --- | --- | --- |
| - | - | - | 1 |  |
| + | - | - | 1.06（0.43-2.64） | 0.894 |
| - | - | + | 0.74（0.30-1.85） | 0.517 |
| - | + | - | 2.81（1.91-4.15） | <0.0001* |
| + | - | + | 1.76（0.16-19.85） | 0.648 |
| + | + | - | 4.71（2.30-9.64） | <0.0001* |
| - | + | + | 8.66（2.95-25.43） | <0.0001* |
| + | + | + | 14.34（1.85-111.19） | 0.011* |

95% CI: 95% Confidence Interval; OR: Odds ratio.

^a^*P* value: Adjusted for sex and age (≤40 and >40 years).

* Statistically significant.
