# Supplementary material for: Anti-Toxoplasma gondii antibodies as a risk factor for the prevalence and severity of systemic lupus erythematosus
Source: Parasit Vectors. 2024 Jan 30;17:44. doi: 10.1186/s13071-024-06141-8 (PMC10826107; doi:10.1186/s13071-024-06141-8)
Supplement: Supplementary file 5 — Additional file 5: Table S5. Risk factors for disease severity (analysis with 3 factors): anti-T. gondii antibodies IgG, anti-dsDNA and anti-rRNP. [file 13071_2024_6141_MOESM5_ESM.docx]

**Table 5** Risk factors for disease severity (analysis with 3 factors): Anti- *T. gondii* antibodies IgG, Anti-dsDNA and Anti-rRNP.

| ATxA-IgG | Anti-dsDNA | Anti-rRNP | | OR（95%CI） | ^a^*P value* |
| --- | --- | --- | --- | --- | --- |
| - | - | | - | 1 |  |
| + | - | | - | 1.05（0.32-3.52） | 0.933 |
| - | + | | - | 3.21（2.01-5.10） | <0.0001* |
| - | - | | + | 1.62（0.83-4.14） | 0.157 |
| + | + | | - | 4.97（2.10-11.77） | <0.0001* |
| + | - | | + | 2.16（0.58-8.08） | 0.252 |
| - | + | | + | 7.08（3.96-12.65） | <0.0001* |
| + | + | | + | 9.00（3.27-29.95） | <0.0001* |

95% CI: 95% Confidence Interval; OR: Odds ratio.

^a^*P* value: Adjusted for sex and age (≤40 and >40 years).

* Statistically significant.
