# Supplementary material for: Anti-Toxoplasma gondii antibodies as a risk factor for the prevalence and severity of systemic lupus erythematosus
Source: Parasit Vectors. 2024 Jan 30;17:44. doi: 10.1186/s13071-024-06141-8 (PMC10826107; doi:10.1186/s13071-024-06141-8)
Supplement: Supplementary file 6 — Additional file 6: Table S6. Risk factors for disease severity (analysis with 3 factors): anti-T. gondii antibodies IgG, anti-dsDNA and ESR. [file 13071_2024_6141_MOESM6_ESM.docx]

**Table 6** Risk factors for disease severity (analysis with 3 factors): Anti- *T. gondii* antibodies IgG, Anti-dsDNA and ESR.

| ATxA-IgG | Anti-dsDNA | ESR | OR（95%CI） | ^a^*P value* |
| --- | --- | --- | --- | --- |
| - | - | - | 1 |  |
| + | - | - | 1.30（0.40-5.51） | 0.719 |
| - | - | + | 1.64（0.95-2.84） | 0.078 |
| - | + | - | 4.59（2.65-7.94） | <0.0001* |
| + | - | + | 2.34（0.79-6.93） | 0.124 |
| + | + | - | 5.78（1.82-18.40） | 0.003* |
| - | + | + | 4.66（2.89-7.49） | <0.0001* |
| + | + | + | 8.00（3.70-17.31） | <0.0001* |

95% CI: 95% Confidence Interval; OR: Odds ratio.

^a^P value: Adjusted for sex and age (≤40 and >40 years).

* Statistically significant.
