# Supplementary material for: Anti-Toxoplasma gondii antibodies as a risk factor for the prevalence and severity of systemic lupus erythematosus
Source: Parasit Vectors. 2024 Jan 30;17:44. doi: 10.1186/s13071-024-06141-8 (PMC10826107; doi:10.1186/s13071-024-06141-8)
Supplement: Supplementary file 7 — Additional file 7: Table S7. Risk factors for disease severity (analysis with 3 factors): anti-T. gondii antibodies IgG, anti-dsDNA and anti-cmDNA. [file 13071_2024_6141_MOESM7_ESM.docx]

**Table 7** Risk factors for disease severity (analysis with 3 factors): Anti- *T. gondii* antibodies IgG, Anti-dsDNA and Anti-cmDNA.

| ATxA-IgG | Anti-dsDNA | Anti-cmDNA | OR（95%CI） | ^a^*P value* |
| --- | --- | --- | --- | --- |
| - | - | - | 1 |  |
| + | - | - | 1.333（0.48-3.74） | 0.585 |
| - | + | - | 4.068（2.72-6.08） | <0.0001* |
| - | - | + | 2.947（0.26-33.42） | 0.383 |
| + | + | - | 5.586（2.70-11.55） | <0.0001* |
| + | - | + | 3.220（0.28-36.67） | 0.346 |
| - | + | + | 4.121（1.27-13.35） | 0.018* |
| + | + | + | 11.352（1.40-92.05） | 0.023* |

95% CI: 95% Confidence Interval; OR: Odds ratio.

^a^*P* value: Adjusted for sex and age (≤40 and >40 years).

* Statistically significant.
